# Book Notices

**Published:** 1855-10

**Authors:** 


### BOOK NOTICES.

American Journal of Pharmacy. Published by Authority of the Philadelphia College of Pharamacy. Edited by Wm. Proctor, Jr., &c., &c. ; Bi-monthly, at $3,00 per annum in advance.

The September number of this valuable periodical is well filled with interesting matter. It deserves the patronage both of physicians and druggists.

The Half-Yearly Abstract of the Medical Sciences: being a practical and Analytical Digest of the contents of the principal British, American, and Continental Medical works, published during the preceding six-months ; together with a series of critical reports on the progress of Medicine and the Collateral Sciences during the same period. Edited by W. H. Ranking, M. D., & C. B. Radcliffe, M. D., of London : Published by Lidnsay, & Blakiston, Philadelphia.

Those who wish for a selection of matter from the principal medical periodicals of Europe and America, once in six months, can find it in the above named work. Each number contains about 300 pages, and it is published at $2,00 per annum.

Transactions of the Medical Associationof Southern Central New York; at the Ninth Annual meeting, held in Elmira, June 5th, 1855.

This is a neatly printed pamphlet of 124 pages, containing the proceedings of the above named Society, interesting reports from several committees, and the annual address of the President, Dr. S. H. French, of Lisle, Broome county, N. Y.

The several papers show a very laudable degree of industry on the part of the members of the Society. We wish, however, that we could see in the Transactions of this and other similar societies more of the results of a well devised system of original experimental researches.

Our medical societies are occupied too much with the mere gathering up of simple isolated cases or disjointed facts. We think more attention should be given to the selection, and investigation of specific questions of interest,—questions, which should lead to investigations calculated to throw additional light on points hitherto obscure and undetermined. It is of but little moment to multiply the records of individual cases of diseases, for instance, unless they present some features calculated to illustrate an unsettled question either in pathology or practice.

---

Braithwaite's Retrospect of Practical Medicine and Surgery; being a half-yearly journal, coutaining a retrospective view of every discovery and practical improvement in the Medical Sciences. Edited by W. BRAITHWAITE, Lecturer on Obstetric Medicine, &c.: Part. 31, July, 1855. American edition, Published by STRINGER & TOWNSEND, 222 Broadway, N. Y., at $2,00 per annum or $1,00 per each number.

THIS, like Ranking's Abstract, furnishes the reader in a cheap form, a very useful summary of the more important matters published in the medical periodical literature of Europe. Each number contains little more than 300 closely printed pages.

---

Discovery of the Cause, Nature, Cure, and Prevention of Epidemic Cholera. By L. M. KNAPP, M. D., late Professor, &c. Published by H. W. DERBY: Cincinnati, 1855.

THIS is the imposing title to a pamphlet or monograph of 48 pages; notices of which we had seen in several of the newspapers and periodicals of the day. These notices with the letters of different persons on the cover, induced us to read the essay at once. All there is essential in its doctrines may be stated in very few words.

The author's discovery consists in the naked declaration that cholera is only a modification of *scurvy*, and arises from a deficient supply and use of *succulent* vegetables. This is the sum total of his discovery of the *nature* and *cause* of the disease. The prevention and cure is of course obvious, viz.: to make free use of such succulent vegetables as are known to prevent and cure *scurvy*.

It is sufficient to say that the positions of the author are not sustained by any collection of carefully observed facts, statistical or otherwise; but on the contrary are made to rest on a series of the most inaccurate, and we might say reckless, general statements that we have ever seen in print. As a specimen of the mode of writing we give the following, viz.: "The reasons drawn from observation, for believing cholera to be of *scorbutic* character, are as follows, to wit: 1st. The coincidences of cold winters and retarded springs preceding the outbreaks. It is a matter of history that the winter of 1831-2 was one of the coldest ever known."

"Again, the winter of 1848-49 was a remarkably cold winter all over the United States, the spring of '49 greatly retarded, and all degrees of the scorbutic diathesis were observed at the bedside by the writer of this, who had for years been an observer of its stealthful, annual appearance. The universal spread of Cholera in the cities of the United States, in the summer of 1849, is well remembered, and its continuance until the coming in and use of the new crops of vegetables and fruits. Their free use, however, during the prevalence of Cholera, has never been known or tolerated. They have rotted in the field rather. It has been held fool-hardy or tempting Providence to eat them.

Casting a glance across the Atlantic, the years 1846-47 were years of dearth, scarcity and blight in Europe, particularly of the potatoe, the most valuable anti-scorbutic vegetable known; and the scurvy and Cholera together followed, scourging the nations generally. Writers in describing the spread of Cholera in Europe in 1847, say, substantially, that it started from India, again, early in that year, passed through the cities of Persia, and thence along the shores of the Caspian, reached Astracan in July, and appeared at Moscow, faint and weary, in the fall, where it took a refreshing sleep through the winter, but woke up in June, 1848, much refreshed and invigorated, and pursued its travels *via* St. Petersburg, Berlin, Hamburg, etc., to Edinburg and London, where it arrived in November *en route* for Paris. It is worthy of remark, that it struck England again, in her weakest and most scorbutic point, her Newcastle coal mines, and foul holds of her coal vessels; and that, prior to its reaching France, the *cause* of it set sail from Havre for America, in two emigrant ships that sailed in October and November. In the October ship it broke out when sixteen days at sea; and the emigrants were landed at Quarantine, in New York, in November, and the disease spread through the Quarantine Hospital, where the sickly, scorbutic inmates were kept on a routine dietary, and whose cup of affliction was made to run over through panic and an increased accumulation of foul air: and a few cases occurred among the emigrants that went to the 'Five Points,' in the city of New York, where, however, it could make no progress, because of the abundance of fall fruits and vegetables; so it again slept through the winter, the intensely cold winter that followed. In the other ship it broke out when twenty-six days at sea, the *cause* in the emigrants having been more subdued before embarkation, by autumnal fruits and vegetables, than in those of the other ship, which sailed a month earlier; and the passengers were landed at New Orleans in December, where the disease spread among emigrants and the poor, the weather, for the season of the year, being hot, and a further reason, potatoes in the south scarce; and it continually appeared on the Mississippi in steamboats, among the emigrant passengers, all winter, and in the emigrant towns as high up as St. Louis and Cincinnati, and established itself gradually in the ports further and further north, as potatoes and succulent vegetable stores failed, and warm, debilitating spring weather came on.

Applying the same theory, the want of succulent vegetable food, to the rather general prevalence of Cholera in the cities of the United States this season, 1854, a rather intense causation is found, of general application, in the extravagantly high prices of provisions during the present year, high prices not only betokening scarcity, but being the same in effect to the poor. I notice the fact in the public prints, that scurvy and Cholera were both found raging simultaneously in the Poor-house at Buffalo this summer.

Thus the coincidences of cold winters and retarded springs, preceding the outbreaks of Cholera in the United States, its vernal appearance, solstitial ragings, autumnal recessions and wintry slumbers, together with its uniform and close communion with scurvy, prove that it is produced and governed by the laws that produce and govern scurvy."

In reference to the correctness of the statement in regard to the *coldness* of the winter of 1831-2, and the alledged scarcity of vegetable food during the succeeding spring, we have not taken the trouble to inquire; but in the remainder of the quotation there are not less than three direct misstatements of facts, besides a self contradiction. In the first place the winter of 1848-49 instead of being "remarkable cold," was actually milder and shorter than the average of winters in the Atlantic States We were then residing in New York city, and remember very well that we had no cold weather until the first of January. But during all the months of November and December, while the Cholera was prevailing in the Quarantine of New York and in the city of New Orleans, the weather was unusually warm and wet. Again, during these months there were *no* cases of Cholera "among the emigrants that went to the '*Five Points*' in the city of New York; nor indeed is there any evidence that a single emigrant went to that section of the city. The first cases that were known to exist on the "Five Points," did not occur until the following May, 1849, when no profusion of succulent vegetables arrested its prevalence until the next autumn.

Again, the winter that preceded the summer of 1854, was as mild and pleasant as any that we have experienced during a residence of six years in Chicago. Neither was the spring of 1854 retarded or backward, for we had a week of quite warm and summer-like weather before the end of April; and there was certainly no lack of vegetables in Chicago during either of the years 1853 or 54. Yet in the latter summer we had one of the severest scourges of cholera that our goodly city was ever afflicted with. The following winter, however, (that of 1854-55) was extraordinarily cold and protracted; the quantity of snow beyond all former precedent; and the prices of both fuel and provisions in this city, fully double that of ordinary seasons. The spring, too, was remarkably cold and backward. And yet during all the summer following the cholera has scarcely been heard of among us. But we are wasting time on this pamphlet. It is the best specimen of that class of productions, where the writer *assumes* a theory and then states his pretended facts in such a way as to correspond without any regard to accuracy, that we have met with.

